# A Two-Step Method Based on $l_{z}^{*}$ for Identifying Effortful Respondents

**DOI:** 10.3390/jintelligence14020030

**Published:** 2026-02-13

**Authors:** Yilan Chen, Yue Liu, Hongyun Liu

**Affiliations:** 1 Faculty of Psychology, Beijing Normal University, Beijing 100875, China; yilanchen@mail.bnu.edu.cn; 2 College of Psychology, Sichuan Normal University, Chengdu 610066, China; helena701@126.com

**Keywords:** person-fit statistic $l_{z}^{*}$, unsupervised learning algorithms, two-step method, effortful respondents

## Abstract

The likelihood-based person-fit statistic, $l_{z}^{*}$, is commonly used in educational assessments to distinguish between respondents who are putting in effort and those who are not. However, $l_{z}^{*}$ depends on the estimated item parameters. Item parameter estimates based on data containing non-effortful respondents are biased, thereby undermining the strength of $l_{z}^{*}$. To address this issue, we propose a two-step method that leverages data mining techniques to obtain more accurate item parameter estimates and then uses them to compute $l_{z}^{*}$. The results show that the estimates based on the effortful group identified by K-means are more accurate, which improves the performance of $l_{z}^{*}$ in terms of the precision of identifying effortful respondents when non-effort severity is high.

## 1. Introduction

The primary goal of educational measurement is to assess the extent to which individuals have acquired knowledge, abilities, and skills. However, the existence of non-effortful response behaviors affects the reliability and validity of assessments, which brings about a series of undesirable consequences. Specifically, examinee effort is typically defined as an individual’s engagement and expenditure of energy toward the goal of attaining the highest possible score on the test (Wise & DeMars, 2005). Conversely, non-effortful response behaviors represent a failure to engage in an assessment. Biased ability estimates resulting from a respondent group mixed with non-effortful individuals can lead to unfair and inappropriate decisions and inferences based on these scores (de la Torre & Deng, 2008). Moreover, non-effortful responses also result in inaccurate item parameter estimates, which can mask response patterns and affect item response theory (IRT) applications (Hong & Cheng, 2019), such as scale linking (van der Linden & Barrett, 2016) and item selection in computerized adaptive testing (Patton et al., 2013). Therefore, how to eliminate the negative effect of unreliable data is an important issue. A straightforward way for mitigating the negative impact is to identify the respondents with non-effortful responses (de la Torre & Deng, 2008), and subsequently adjust the weight of corresponding responses, even to zero.

### 1.1. Traditional Approaches and Challenges

The approaches to detecting non-effortful respondents can be divided into two categories. The first category inserts specialized items into the test prior to administration. One version of these items covertly attempts to flag the respondents who carelessly read the item stem, or to index respondent care in response, such as bogus items and instructed response items. A second version involves the use of self-report measures administered after the test has been completed. Respondents are required to report their overall performance on the test rather than the details of every single item (Meade & Craig, 2012). The second category can be described as post hoc methods, which involve special analyses after data collection is complete. These methods include longstring, outlier analysis, person-fit statistics, and mixture models, which focus on analyzing responses (and response times) (Drasgow et al., 1985; Meyer, 2010; Ranger & Kuhn, 2017). Ward and Meade (2023) offer an overview of various detection methods, and Curran (2016) provides details on the computation.

Among the approaches mentioned above, person-fit analysis is widely used (Yavuz Temel et al., 2022) and expanding in research (Gorney & Wollack, 2022b; Tong et al., 2022). It measures the degree of fit between an observable pattern and the theoretical model (de la Torre & Deng, 2008). Methods in this category are a diverse group and can be further broken down into two categories: nonparametric and parametric person-fit statistics. Many nonparametric person-fit statistics (e.g., U3; van der Flier, 1982; *H*^T^; Sijtsma, 1986) are founded on the concept of Guttman errors (Beck et al., 2018). A Guttman error occurs when a respondent answers an easy item incorrectly but a more difficult item correctly. In practice, the simple or normed count of Guttman errors is often used as a straightforward method for detecting non-effortful respondents. Parametric person-fit statistics can be thought of as a more sophisticated version of Guttman errors (Ward & Meade, 2023). Compared with nonparametric person-fit statistics, most of which are not based on estimated model parameters, they can easily incorporate more factors that influence response patterns, such as accounting for latent classes via a mixture IRT model. The index $l_{z}$ is one of the most popular in this category (Lee et al., 2024; Min & Aryadoust, 2021; Sinharay, 2017) due to its ease of computation and comparison. However, the comparison between observations and models relies on the asymptotic assumption (Magis et al., 2012). This assumption is conditional on the known ability parameters. When sample estimates are used to replace these parameters, the variance of $l_{z}$ is shrunk (Hong & Cheng, 2019), which means that the assumption cannot be satisfied (de la Torre & Deng, 2008). As a consequence, $l_{z}$ may detect non-effortful respondents in an overly conservative way, leading to a lower TypeIerror rate and power (Gorney & Wollack, 2022a).

For the Rasch model, the influence of the unknown ability parameter on the distribution of $l_{z}$ can be neutralized by conditioning on the total score, because the total score is a complete sufficient statistic for the ability parameter. I. W. Molenaar and Hoijtink (1990) suggested two methods to approximate the null distribution of test statistics like $l_{z}$ for the dichotomous Rasch model. The first method is based on the Cornish-Fisher expansion, and the other is the chi-square approximation. von Davier and Molenaar (2003) proposed an extension of the work of I. W. Molenaar and Hoijtink (1990) to mixture distribution IRT models for polytomous categorical data. Bedrick (1997) proposed a different approximation based on an Edgeworth expansion.

However, they did not deal with the unknown nature of the ability parameter. Snijders (2001) offered a solution. The author corrected the weight function used in the calculation of $l_{z}$. The corrected $l_{z}$ is referred to as $l_{z}^{*}$. After the correction, the functions of $\theta_{i}$ are approximately equal to those of ${\hat{\theta }}_{i}$. However, Snijders (2001) pointed out that these approximations only make sense when the uncertainty of estimated item parameters is relatively small compared to that of the unknown ability parameter. Thus, $l_{z}^{*}$ may still be biased due to the large bias of item parameter estimates based on the mixture sample, even though the performance of the corrected statistic improved to some extent. Patton et al. (2019) found that the maximum bias (absolute value) of the discrimination parameter increased from 1.5 to 2.5, and that of the location parameter from 0.3 to 0.4, the resulting power of $l_{z}^{*}$ decreased from 0.96 to 0.76, as the percentage of non-effortful respondents increased from 10% to 30%. To improve the performance of $l_{z}^{*}$, obtaining accurate item parameter estimates based on an effortful respondent sample is imperative (Patton et al., 2019).

Gorney et al. (2024) proposed three new corrections for the class of standardized person-fit statistics (e.g., $l_{z}$) that simultaneously account for the use of a finite number of items and the use of an estimated ability parameter. However, their approach also relies on an assumption common in person-fit research—that the item parameters are treated as known.

Because the correct response probability from non-effortful responding usually exhibits a different pattern than effortful responding, quite a few mixture models have been proposed to explicate different data-generating processes associated with response behaviors. For instance, Boughton and Yamamoto (2007) propose a HYBRID model that can detect respondents who have switched to random responses. Their model assumes that responses follow the Rasch model until a point at which respondents have switched from an ability-based response strategy to a rapid guessing or random response strategy. Wang and Xu (2015) propose a mixture hierarchical model to distinguish between solution and rapid guessing behaviors for each item. The model includes a latent variable indicating response behavior. Wang et al. (2018b) replace the person-level guessing propensity parameter ($\pi_{i}$) in Wang and Xu’s model by an item-level parameter ($\pi_{j}$). Ulitzsch et al. (2020) propose a mixture model to identify disengagement at the item-by-person level. Their model assumes different data-generating processes underlying item omissions and responses, as well as response times associated with engagement and disengagement. Dependent latent class IRT (DLC-IRT) modeling is a recent innovation proposed by Nagy and Ulitzsch (2021). Unlike the most commonly used IRT models (Ulitzsch et al., 2020; Wang & Xu, 2015; Wang et al., 2018b), it treats response times as predictors of response engagement. Similar to $l_{z}^{*};$ however, most of these methods are restricted to particular assumptions. For example, the mixture model method assumes that the vector consisting of response time for each item follows the multivariate log-normal distribution given the particular latent class of respondents. The violation can result in biased parameter estimates. Therefore, data mining can be considered as an alternative approach, since it is free of strong assumptions.

### 1.2. Data Mining Methods as Alternatives

Data mining has drawn dramatic attention across various scientific areas. An increasing number of applications have expanded in psychological and educational fields, such as student classification (Goldhammer et al., 2017). This technique has shown surprising potential in the identification of different types of response behaviors (Miller et al., 2016; Sinharay, 2016) due to its ability to simultaneously examine both linear and nonlinear relationships among variables, without strong assumptions (Man et al., 2019). In comparison, traditional manners, constrained by assumptions, only allow for a specific relationship via mixture models for response and response time, and are usually confronted with non-convergence problems caused by model complexity (Lu et al., 2021; D. Molenaar et al., 2018; Pokropek, 2016). Man et al. (2019) compared several data mining methods with person-fit statistics, including $l_{z}^{*}$. The results demonstrated that both supervised and unsupervised methods had higher identification rates of non-effortful respondents than the traditional method.

Unsupervised learning algorithms may be more feasible than supervised learning algorithms, although the former have usually been applied to a single empirical data set (i.e., one specific scenario) (Doval & Delicado, 2020; Lundgren & Eklöf, 2021; Man et al., 2019; Qiao & Jiao, 2018) rather than being evaluated thoroughly under various conditions. This is because training sets used in supervised learning may be invalid. Specifically, researchers who assign labels to respondents in training sets are uncertain about which respondents are non-effortful (Patton et al., 2019).

By contrast, unsupervised methods are utilized when respondents’ memberships are unknown, which makes them a more practical approach to address such educational measurement issues. Cluster analysis and self-organization mapping (SOM; Kohonen, 1982) are two well-established unsupervised techniques that recognize response patterns (Qiao & Jiao, 2018). For instance, Liao et al. (2021) proposed a two-step approach utilizing K-means to learn archetypes of the responding process and identify respondents with abnormal behavioral patterns, applying it to an empirical dataset. Specifically, archetypes of responding processes were learned based on response times, the proportion of actions involving initial item visit, and the number of answer changes. Subsequently, behavioral archetypes were labeled as codewords, of which the frequencies were used as features for respondent-level K-means clustering. Soller and Stevens (2007) applied SOM to categorize respondents’ problem-solving strategies. In their study, input vectors described sequences of respondent actions during problem solving. The training result was a topological ordering of nodes according to the structure of the data, where the distance between nodes reflected the similarity of respondents’ strategies. Fossey (2017) evaluated K-means, SOM, and Robust Clustering Using Links (ROCK) on grouping response patterns based on logfile data in a game-based assessment scenario. Given the feasibility and empirical performance of unsupervised learning algorithms, it is worthwhile to further investigate the methods’ identification patterns. In the current study, we chose the first two unsupervised learning algorithms, K-means clustering and SOM, to identify an effortful group for more accurate item parameter estimates, since ROCK is not directly applicable to continuous data, such as response time, which serves as a primary source of evidence for effortful responses.

K-means clustering is the most commonly used partitional clustering algorithm in various domains (Jain, 2010; Khan & Ahmad, 2004). Researchers (Doval & Delicado, 2020; Lundgren & Eklöf, 2021; Man et al., 2019) employ this algorithm to flag students as different types, such as cheaters, non-effortful responders, and so on.

SOM is a widely used tool for grouping and visualizing observations in scientific areas. It is an artificial neural network algorithm (Wehrens & Kruisselbrink, 2018), proposed by Kohonen (1982). Similar to K-means (Goldhammer et al., 2017; H. Y. Liu, 2019; Qiao & Jiao, 2018), SOM is commonly used to classify individuals and select features in psychological research (Man et al., 2019; Qiao & Jiao, 2018).

The reasons why we chose two methods can be summarized as follows. K-means is easy to understand and simple for practitioners to implement without complex parameter specification and model construction. It can be implemented through most data analysis software, such as SPSS and R. Additionally, it is computationally efficient due to the nonparametric distance measure, even when handling high-dimensional data, such as complex process data collected in digital assessments. This measure consumes less computational memory than parametric ones (Hastie et al., 2009). As for SOM, it can clearly project complex topological relations in a high-dimensional data set to a two-dimensional grid. Moreover, SOM is less influenced by tiresome outliers (Wehrens & Buydens, 2007). It moves cluster centers based on the case considered at every iteration rather than by averaging points assigned to the same clusters (Man et al., 2019). In addition to the characteristics of algorithms themselves, both have been used to identify individual response patterns or latent constructs reflected by these patterns (e.g., problem-solving strategies).

In this article, we propose a two-step method that combines the two unsupervised algorithms with $l_{z}^{*}$. K-means and SOM are used to improve item calibration in the first identification, and $l_{z}^{*}$ is computed based on the estimated parameters in the next step.

The rest of this article is organized as follows. First of all, we provide an overview of the approaches (i.e., K-means, SOM, and $l_{z}^{*}$) involved in the two-step method and illustrate the procedure of the proposed method. Then, we evaluate the performance of the method in a simulation study by calculating recall, precision, and *F*1 of effortful respondents. Next, we apply the proposed method to real data collected from undergraduates in an American university (Pastor et al., 2019). Finally, we conclude with a discussion on the method’s detecting power and direction for future research.

## 2. Methods

### 2.1. The $l_{z}$ Statistic

The $l_{z}$ statistic was defined as (Drasgow et al., 1985)

(1) $$l_{zi}=\frac{\sum_{j=1}^{J}w_{ij}\left(u_{ij}-P_{j}\left(\theta_{i}\right)\right)}{\sqrt{\sum_{j=1}^{J}w_{ij}^{2}P_{j}\left(\theta_{i}\right)\left(1-P_{j}\left(\theta_{i}\right)\right)}},$$

where *i* and *j* index the person and item, respectively, and *J* is the test length. Here, $w_{ij}$ is a weight function

(2) $$w_{ij}=\log\left(\frac{P_{j}\left(\theta_{i}\right)}{1-P_{j}\left(\theta_{i}\right)}\right),$$

$u_{ij}$ is 0 or 1 according to whether person *i* responds to item *j* correctly for dichotomous items, $\theta_{i}$ is the ability parameter of person *i*, $P_{j}\left(*\right)$ denotes the probability of person *i* answering item *j* correctly, in a two-parameter logistic (2PL) model (Birnbaum, 1968)

(3) $$P_{j}\left(\theta_{i}\right)=\frac{\exp\left(a_{j}\left(\theta_{i}-b_{j}\right)\right)}{1+\exp\left(a_{j}\left(\theta_{i}-b_{j}\right)\right)},$$

where $a_{j}$ and $b_{j}$ are the discrimination and difficulty parameters of item *j,* respectively. According to $l_{z}$ asymptotically following the standard normal distribution, examinees whose $l_{z}$ < −1.645 are flagged as non-effortful respondents at the 0.05 significance level.

The weight function is corrected for the sampling variability (Snijders, 2001),

(4) $$w_{ij}^{*}=w_{ij}-c_{J}\left({\hat{\theta }}_{i}\right)r_{j}\left({\hat{\theta }}_{i}\right),$$

where

(5) $$c_{J}\left(\theta_{i}\right)=\frac{\sum_{j=1}^{J}P_{j}^{\prime }\left(\theta_{i}\right)w_{ij}}{\sum_{j=1}^{J}P_{j}^{\prime }\left(\theta_{i}\right)r_{j}\left(\theta_{i}\right)},$$

$P_{j}^{\prime }\left(\theta_{i}\right)$ is the first-order derivative with respect to θ, and

(6) $$r_{0}\left({\hat{\theta }}_{i}\right)+\sum_{j=1}^{J}\left[u_{ij}-P_{j}\left({\hat{\theta }}_{i}\right)\right]r_{j}\left({\hat{\theta }}_{i}\right)=0.$$

Various estimators of ability (e.g., the maximum likelihood estimator, Warm’s (1989) weighted likelihood estimator, and Bayesian posterior mode estimators) and different IRT models could be employed to compute the extension of $l_{z}$. Note that $r_{0}\left(\theta_{i}\right)$ and $r_{j}\left(\theta_{i}\right)$ rely on estimation methods. It is in the maximum likelihood estimation that $r_{0}\left(\theta_{i}\right)=0$ and $r_{j}\left(\theta_{i}\right)$ is defined as the derivative of the log-odds in Equation (2). The slightly modified index is referred to as $l_{z}^{*}$.

### 2.2. K-Means Clustering

This method consists of the following five steps:

1. Choose *K* initial centers (i.e., means of clusters) that can be randomly selected from data or defined by researchers. The number of clusters, *K*, can be determined based on the researchers’ hypotheses about response patterns or through statistical techniques. Various statistical methods are available for determining the optimal *K*, such as the “elbow methods” (Thorndike, 1953), silhouette width (Rousseeuw, 1987), and the Dunn index (Dunn, 1974).
2. Calculate the distance between each point and each center in turn, and then assign all the points to their closest centers. The distance measure can also be user-specified, such as the Euclidean distance and Manhattan distance.
3. Recalculate the new centers.
4. Repeat steps 2 and 3 until clusters do not change, that is, no point switches between clusters.
5. The definition and interpretation of clusters are mainly based on response accuracy, response time, or both. For instance, the cluster with a higher average response time is more likely to be defined as the effortful group, whereas the other is more likely to be defined as the non-effortful group. This is supported by the characteristic of rapid guessing, which is typical of the non-effortful group. Specifically, rapid guessing has a shorter response time (Wise, 2015).

### 2.3. Self-Organization Mapping (SOM)

**Figure 1:**
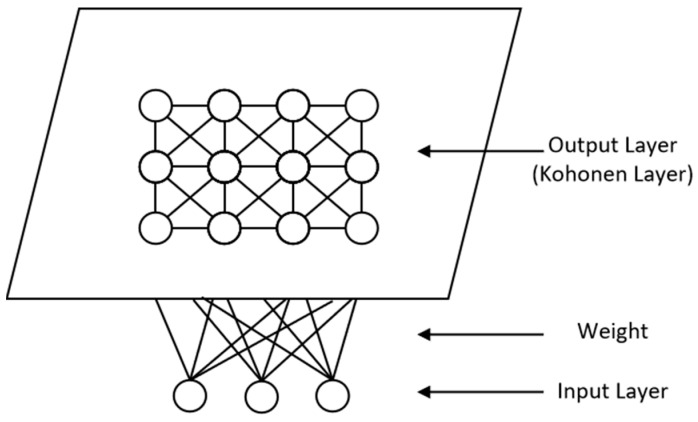
Illustration of SOM.

Compared with K-means, which clusters directly data points in a high-dimensional space, SOM, as shown in Figure 1, aims to map multidimensional data to a two-dimensional plane while maintaining the same topological order, and makes similar ones closer while the dissimilar ones are farther away from each other through weight adjustment (Wehrens & Kruisselbrink, 2018). This is accomplished in several steps (Kohonen, 1997):

1. Initialize and normalize weights that connect the input layer and output layer. The initialization assigns small random numbers to the weight.
2. Calculate the distance between the randomly selected input neuron and all output neurons. Input neurons represent data points, while output neurons are the corresponding nodes to which the observed points map. There are more than two nodes in the output layer, whose lattice type is usually hexagonal or rectangular.
3. Choose the closest output neuron as the best matching unit (BMU).
4. Update the weight of the BMU and its neighbor neurons to make them more sensitive to similar input signals.
5. Repeat steps 2 to 4 until the radius of the neighborhood decreases to zero.
6. The group types are determined in the same manner as K-means.

### 2.4. The Two-Step Method

To improve the performance of $l_{z}^{*}$ in data sets mixed with non-effortful respondents, we propose a two-step method. This method involves two times identification, the first of which is to obtain the effortful data used in a more accurate estimation of item parameters. The procedure is as follows:

1. Use K-means or SOM to enact clustering (i.e., the first identification).
2. Estimate item parameters based on the effortful cluster.
3. Using the above estimates, compute $l_{z}^{*}$ for all the respondents. Alternatively, only respondents with the non-effortful label in the first identification are identified for the second time. This is because the non-effortful respondents ignored by K-means and SOM in the first identification are possibly undetected by $l_{z}^{*}$ in the second identification. The non-effortful respondents, these three methods distinguished, are similar due to the similar identification strategy (i.e., based on the similarity between respondents). Man et al. (2019) found that compared with traditional person-fit statistics, the unsupervised learning algorithms would likely correctly flag a larger percentage of non-effortful respondents at the price of a lower identification rate of normally behaved respondents. So, the non-effortful identified by $l_{z}^{*}$ are possible to be included in the non-effortful group identified by the other methods.
4. The respondents with $l_{z}^{*}$ > −1.645 are assigned to the effortful group. Or merge respondents in the initial effortful cluster, and with $l_{z}^{*}$ > −1.645 into the final effortful group.

According to the respondents who should be identified by $l_{z}^{*}$, there are two versions of the two-step method. One version re-identifies all the respondents, while the other version only re-identifies the non-effortful respondents flagged in the first identification.

## 3. Simulation Studies

### 3.1. Design

A pre-study was conducted to investigate the identification patterns of K-means, SOM, and $l_{z}^{*}$. These results served as the potential for combining the former two methods with $l_{z}^{*}$. The main study was conducted to investigate the difference between the two versions of the proposed method and its viability, that is, whether data mining can improve the performance of $l_{z}^{*}$ in the identification of effortful respondents.

Pre- and main studies were conducted under the same simulation settings. We set up simulation scenarios following (Y. Liu et al., 2020; Wang et al., 2018a, 2018b), while additionally incorporating two factors: sample size (*I*) and test length (*J*). The simulated test consisted of multiple-choice items with four options. Three factors manipulated in previous studies were non-effort prevalence, non-effort severity, and the difference in time spent between effortful and non-effortful respondents. Non-effort prevalence (π) was defined as the proportion of non-effortful respondents. Non-effort severity ($\pi_{i}$) was the proportion of non-effortful responses for respondent *i*. Table 1 presents the levels of each factor. Sample size was varied at three levels (500, 1000, 2000). The test length featured three levels (15, 30, 50). Prevalence had two levels: 20% and 40%. Severity varied between two levels: low ($\pi_{i}$ was drawn from a uniform distribution *U* (0, 0.25)) and high ($\pi_{i}$ was drawn from *U* (0.5, 0.75)). The time difference was manipulated by setting different non-effortful response times. We generated the logarithmized non-effortful response time from normal distribution *N* (−1, 0.25) or *N* (−2, 0.25) while the logarithmized effortful response time from the normal distribution controlled by latent speed parameters and time-related parameters (with details given below). For each condition, 200 replications were performed.

### 3.2. Data Generation

The effortful data were generated based on a hierarchical model (van der Linden, 2007).

The first level contains the response accuracy model and the response time model. In this study, the effortful response and response time (RT) for each item were simulated from the 2PL model and the lognormal model, respectively,

(7) $$\left\{\begin{array}{c} P_{j}\left(\theta_{i}\right)=\frac{\exp\left(a_{j}\left(\theta_{i}-b_{j}\right)\right)}{1+\exp\left(a_{j}\left(\theta_{i}-b_{j}\right)\right)}\;\;\;2\mathrm{PL}\;\mathrm{model}\\ \log\left(t_{ij}\right)\left|\tau_{i}\right.\;\sim \;N\left(\beta_{j}-\tau_{i},\alpha_{j}^{-2}\right)\;\;\;\mathrm{RT}\;\mathrm{model} \end{array}\right.,$$

where $P_{j}\left(\theta_{i}\right)$ refers to the probability of person *i* with the ability parameter $\theta_{i}$ answering item *j* correctly, $a_{j}$ and $b_{j}$ are discrimination and difficulty parameters for item *j* individually; $t_{ij}$ is the time that person *i* spends on item *j*, $\tau_{i}$ represents the latent speed parameter for person *i*, $\alpha_{j}$ and $\beta_{j}$ are time-related discrimination power and time intensity parameters for item *j*.

The second level contains a joint distribution of person parameters,

(8) $$\xi_{i}\;\sim \;N\left(\mu ,\Sigma \right),$$

where $\xi_{i}$ is the person parameter, $\xi_{i}={\left(\theta_{i},\tau_{i}\right)}^{\prime }$, μ and Σ are the mean vector and covariance matrix, $\mu ={\left(\mu_{\theta },\mu_{\tau }\right)}^{\prime }$, $\Sigma =\left[\begin{array}{cc} \sigma_{\theta }^{2} & \sigma_{\theta \tau }\\ \sigma_{\tau \theta } & \sigma_{\tau }^{2} \end{array}\right]$.

These person parameters were randomly drawn from the distributions shown in Table 2 to mimic real data (Y. Liu et al., 2020; Wang & Xu, 2015). The correlation between $\theta_{i}$ and $\tau_{i}$ was fixed at a moderate level, indicating that high-ability individuals respond faster than low-ability ones (Wang et al., 2018b).

To generate mixture data sets, respondents were randomly selected as non-effortful ones in light of non-effort prevalence and severity (as denoted by π and $\pi_{i}$ in Table 1). Y. Liu et al. (2020) indicated that respondents with low speed are more likely to give a non-effortful response, and this could occur on any item. In accordance with the previous research (Wang et al., 2018b), we drew 60% of non-effortful respondents from the individuals whose true speed parameters were in the lowest one-third, 30% from the middle one-third, and 10% from the upper one-third. While various non-effortful response patterns exist (see, e.g., Glas & Dagohoy, 2007; Jin et al., 2017), we focus exclusively on rapid guessing as it is widely observed across assessments. Given that the correct response probability arising from rapid guessing behavior should be independent of the measured construct, we set it at 0.25, corresponding to the chance level of multiple-choice items with four options (Wang & Xu, 2015). Consequently, only two response patterns, one being effortful and the other non-effortful, are involved in this study.

### 3.3. Analysis

In the pre-study, data sets were analyzed with K-means, SOM, and $l_{z}^{*}$ individually. In the main study, the two-step method was used. K-means, SOM, and $l_{z}^{*}$ were implemented by R using the function kmeans(), the package kohonen (Wehrens & Kruisselbrink, 2018), and the package PerFit (Tendeiro et al., 2016), respectively. The item parameters required for the two-step method were estimated using the R package mirt (Chalmers, 2012).

The initial centers in the K-means algorithm were chosen by default. The number of clusters, *K*, was set at 2 to partition respondents into the effortful group and non-effortful group, aligning with the true number of groups in data generation. Following previous studies (Fossey, 2017; Liao et al., 2021), we use Euclidean distance.

For the SOM analysis, the default distance measure was employed. Specifically, the Tanimoto distance is used for class membership matrices or factors, and the sum-of-squares distance in all other cases. For single-layered maps, the sum-of-squares distance is equivalent to the Euclidean distance (Wehrens & Kruisselbrink, 2018). Based on the distance that represents the similarity of neurons, the output layer was divided into two sections corresponding to the two types of respondents.

In this study, the definition of clusters was based on response times, as they associated with rapid guessing are assumed to be shorter than those associated with effortful responding.

### 3.4. Evaluation Criteria

Recall, precision, and *F*1-score were used to evaluate the identification accuracy of the methods. Specifically, recall was defined as the proportion of correctly identified effortful respondents in the true effortful group, while precision referred to the percentage of correctly identified effortful respondents in the identified effortful group. *F*1-score, the harmonic mean of recall and precision, was adopted for the comprehensive evaluation. For these classification metrics, summary statistics were averaged across replications.

In the pre-study, absolute bias and mean squared error (*MSE*) of item parameter estimates were evaluated to demonstrate whether data mining methods could enhance the identification accuracy of $l_{z}^{*}$ by improving calibration. Since item parameter estimation occasionally yielded extreme outliers that inflated the mean, the median was adopted to summarize the overall parameter recovery.

### 3.5. Results

Within each figure, columns represent the level of prevalence (π) and severity ($\pi_{i}$) of non-effortful responses, while rows correspond to sample sizes (*I*). The *x*-axis denotes the test length (*J*).

**Figure 2:**
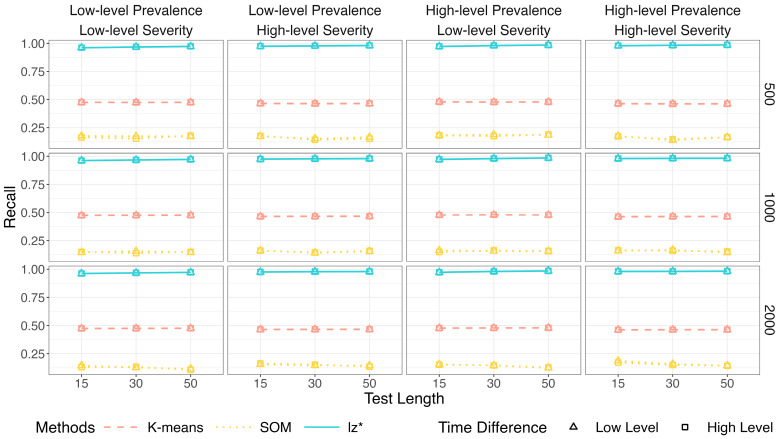
Recall of data mining methods and $l_{z}^{*}$.

**Figure 3:**
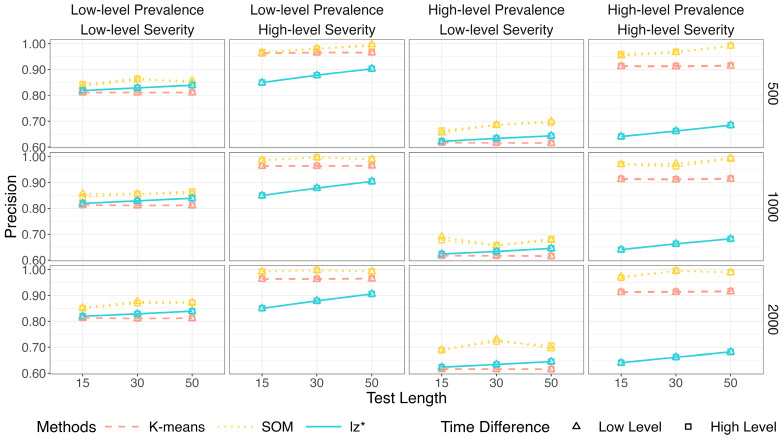
Precision of data mining methods and $l_{z}^{*}$.

**Figure 4:**
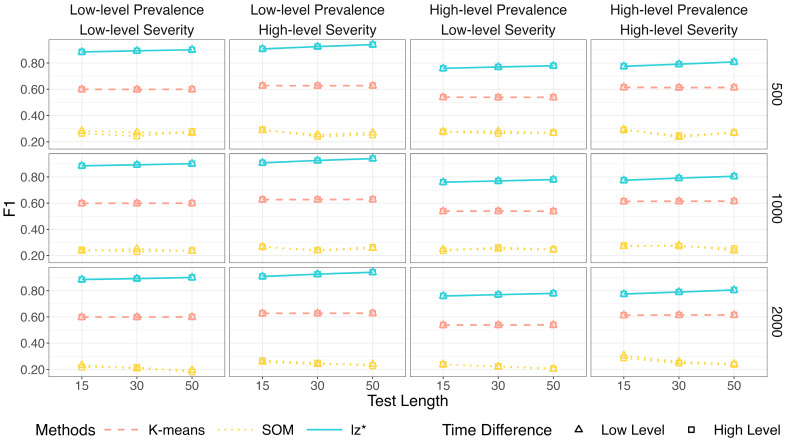
*F*1 of data mining methods and $l_{z}^{*}$.

Figure 2, Figure 3 and Figure 4 show the identification accuracy of K-means, SOM, and $l_{z}^{*}$. The performance patterns of these methods were consistent across different sample sizes and time difference conditions. Figure 2 presents their recall. K-means and SOM correctly identified less than half of the effortful respondents across all the conditions, whereas the recall of $l_{z}^{*}$ ranged from 0.96 to 0.99. Figure 3 shows their precision. Generally, the precision improved slightly as the test length increased. The data mining methods exhibited satisfactory precision when the non-effort severity was high (i.e., $\pi_{i}\;\sim \;U\;\left(0.5,\;0.75\right)$; as shown in the second and fourth columns of Figure 3). In contrast, all the methods displayed similar performance under low-level non-effort severity (i.e., $\pi_{i}\;\sim \;U\left(0,0.25\right)$; as shown in the first and third columns of Figure 3). Figure 4 presents the *F*1-scores of the three methods. Similar to the recall, $l_{z}^{*}$ consistently outperformed K-means and SOM. K-means showed moderate performance, while SOM performed poorly.

**Figure 5:**
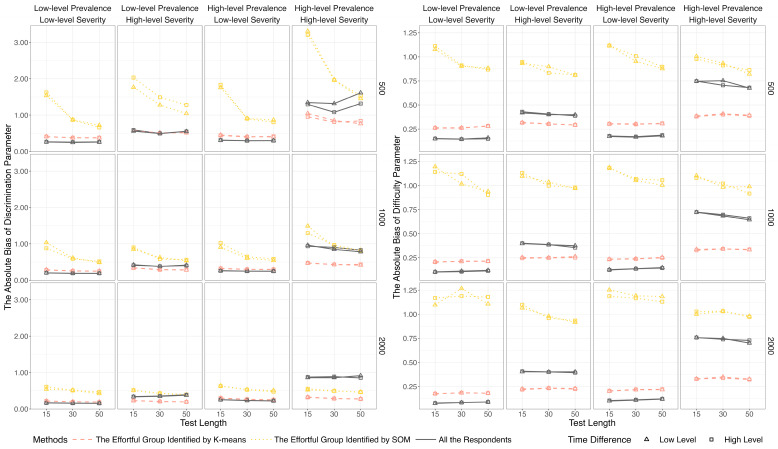
Absolute bias of discrimination and difficulty parameter estimates based on data mining methods and $l_{z}^{*}$.

**Figure 6:**
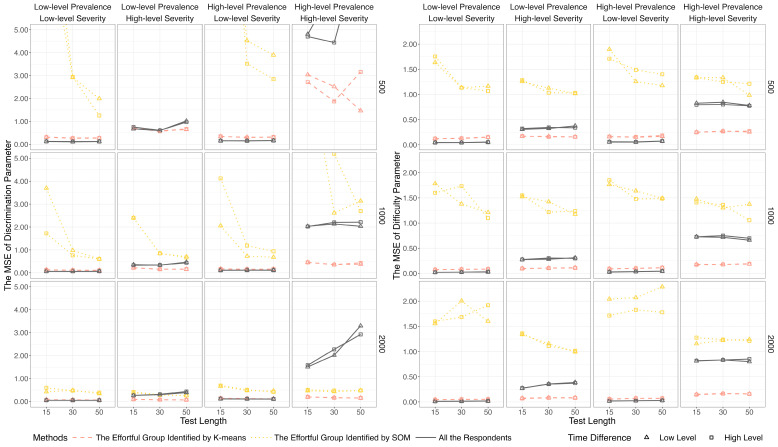
*MSE* of discrimination and difficulty parameter estimates based on data mining methods and $l_{z}^{*}$. *Note.* To clearly visualize the results, the range of the y-axis was restricted. The complete results for the conditions of *I* = 500 and 1000 are provided in Supplementary Tables S1 and S2.

Figure 5 and Figure 6 display the results of item parameter recovery based on the full sample (i.e., all respondents) and effortful groups identified by the data mining methods. A notable pattern is that the estimates based on SOM tended to be severely biased, particularly when the test length was short (i.e., 15 items). Given its poor performance, SOM was excluded from the subsequent investigation. In contrast, K-means showed consistent performance across all conditions, whereas the estimation accuracy based on the full sample was influenced by the level of non-effort severity. Specifically, under the low-severity condition, absolute biases and *MSE*s based on K-means were marginally higher than those based on the full sample. However, under the high-severity condition, parameter estimates based on the whole sample were more biased than those based on K-means.

The findings of the pre-study suggest that utilizing K-means can potentially enhance the identification accuracy of $l_{z}^{*}$ by providing a relatively pure data set for parameter estimation in scenarios with high non-effort severity. However, caution is needed when combining K-means with $l_{z}^{*}$ due to its low recall. It should be noted that the effortful group identified by K-means may not be fully representative of all the effortful respondents, particularly in scenarios with low non-effort severity. As a result, the parameter estimates may still be biased.

**Figure 7:**
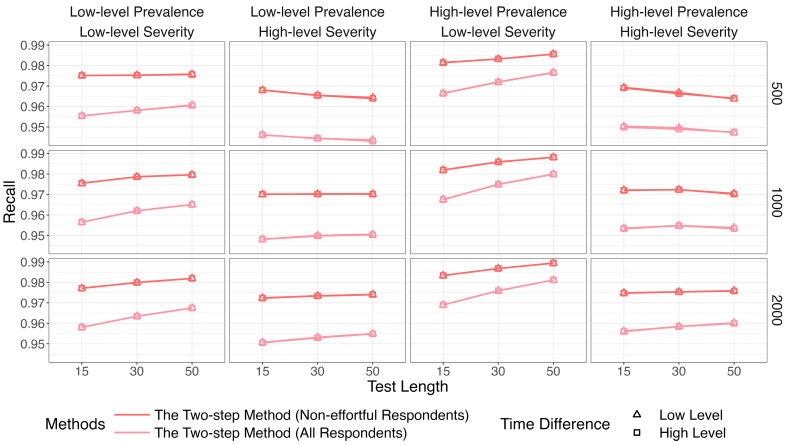
Comparison of recall between the two versions of the two-step methods.

**Figure 8:**
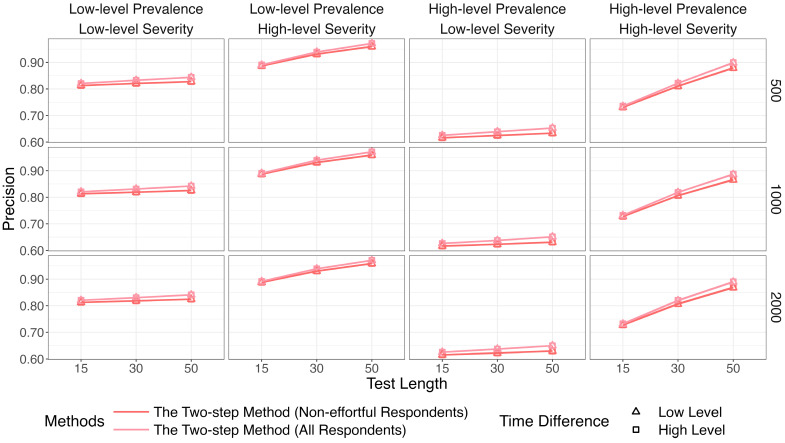
Comparison of precision between the two versions of the two-step methods.

**Figure 9:**
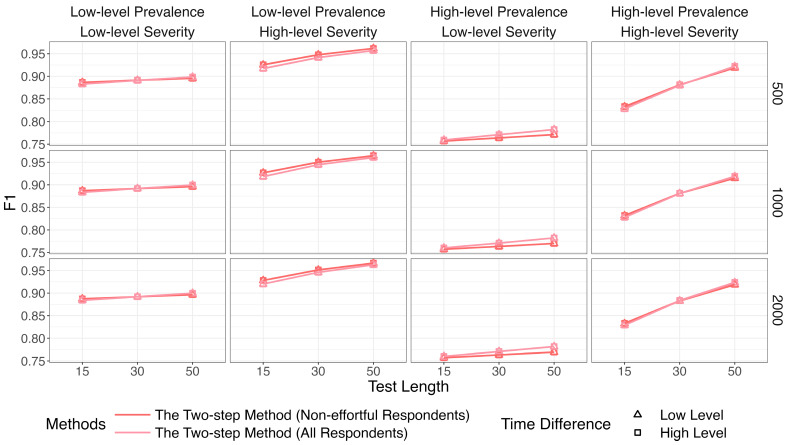
Comparison of *F*1 between the two versions of the two-step methods.

Figure 7, Figure 8 and Figure 9 show that the identification accuracy of the two versions of the proposed method was comparable. For computational efficiency, we recommend calculating $l_{z}^{*}$ for only the respondents who are assigned to the non-effortful group by K-means. We will focus on the performance of the more efficient version in the following sections.

**Figure 10:**
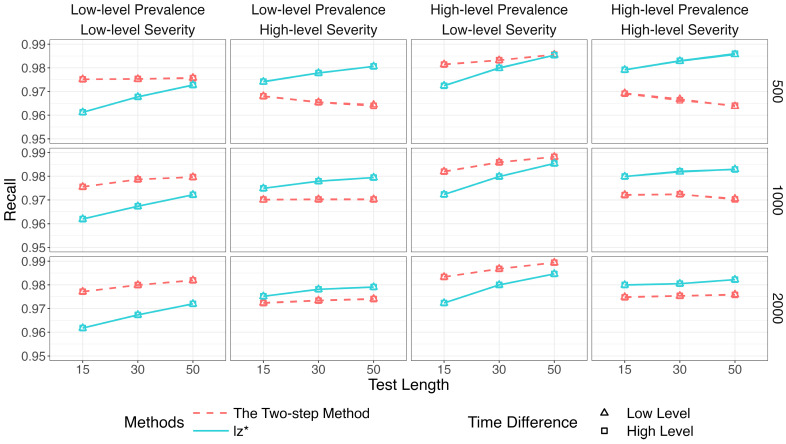
Recall of the two-step method and $l_{z}^{*}$.

**Figure 11:**
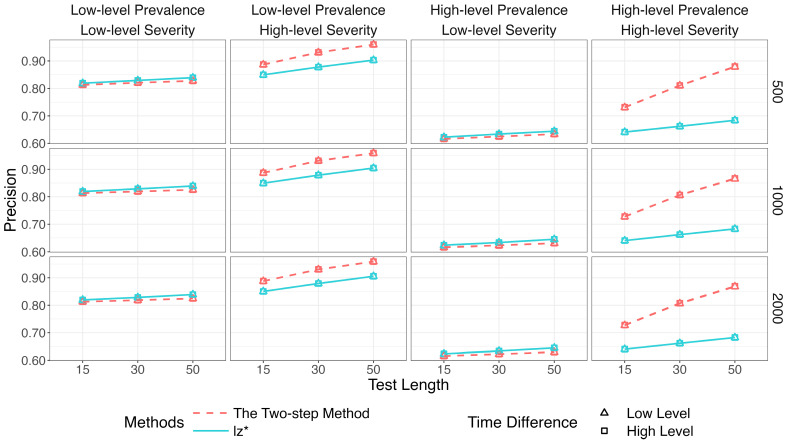
Precision of the two-step method and $l_{z}^{*}$.

**Figure 12:**
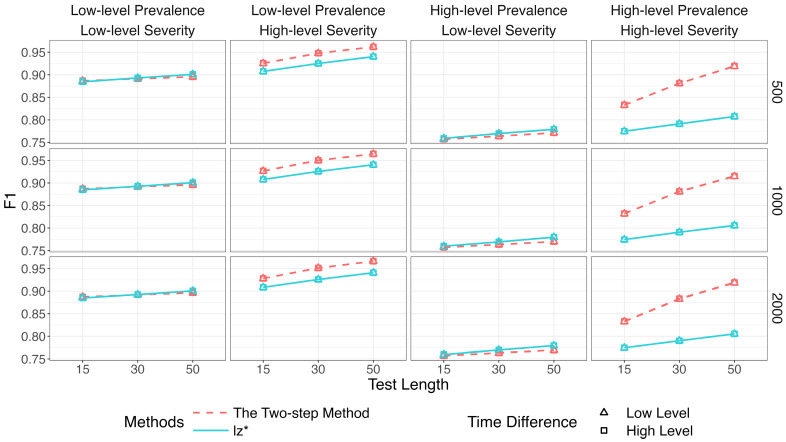
*F*1 of the two-step method and $l_{z}^{*}$.

Figure 10, Figure 11 and Figure 12 compare the two-step method with $l_{z}^{*}$.^1^ Figure 10 depicts results for the recall. The two-step method and $l_{z}^{*}$ remained relatively steady (above 96%) across all conditions. Results for the precision are in Figure 11. The purity of the effortful group was improved when $\pi_{i}$ increased. The two-step method obviously surpassed the traditional way when both π and $\pi_{i}$ were high. The results of the two methods were similar in other conditions.

In sum, when non-effort severity was high, K-means improved the performance of $l_{z}^{*}$ by providing more accurate estimates of item parameters.

## 4. Empirical Example

Except for the simulation studies, the two-step method was used for the analysis of a real data set containing 1532 students in a mid-sized southeastern university. The students participated in an online low-stakes assessment of the natural world during an institution-wide Assessment Day. This assessment consisted of 50 items, and each student’s responses and response times on these items were recorded (Pastor et al., 2019). For data preprocessing, we deleted all records with missing values; 996 students remained.

**Figure 13:**
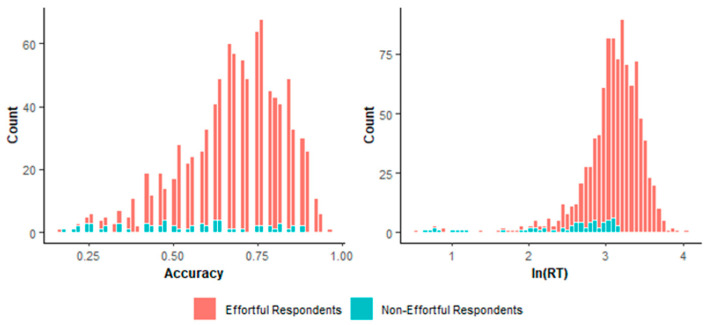
Distribution of accuracy and logarithmized average response time based on the two-step method.

**Figure 14:**
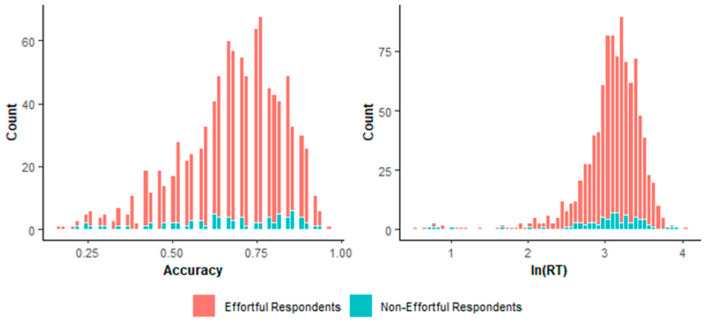
Distribution of accuracy and logarithmized average response time based on $l_{z}^{*}$.

To investigate the performance of the two methods, we compared effortful and non-effortful respondents’ distributions in terms of response accuracy and time. Figure 13 presents the distributions of respondents identified as effortful or non-effortful respondents by the two-step method, while Figure 14 presents those identified by $l_{z}^{*}$. The two-step method identified more respondents with relatively high accuracy and long response time as effortful ones, than $l_{z}^{*}$ did. As truly effortful respondents are generally expected to exhibit higher accuracy and invest more time than non-effortful respondents (Wise, 2017), the two-step method appears to produce more reasonable classifications than $l_{z}^{*}$.

## 5. Discussion

From the psychometrics perspective, the existence of non-effortful responses can be detrimental to item parameter estimation and lead to invalid inferences based on test scores (Wang et al., 2018b). $l_{z}^{*}$ is a common method for distinguishing effortful respondents from a mixed group that includes aberrant respondents. However, the accuracy of $l_{z}^{*}$ can be affected by non-effort prevalence and severity.

In this article, we proposed the two-step method to mitigate the impact of non-effort responses on $l_{z}^{*}$ by improving the accuracy of item parameter estimation. To evaluate the performance of the two-step method, we conducted simulation studies comparing it with $l_{z}^{*}$ used alone.

In the pre-study, we investigated the feasibility of data mining methods to improve item parameter estimates by purifying the calibration sample. The results showed that K-means yielded the most accurate estimates when non-effort severity was high. In contrast, the performance of SOM was far from satisfactory, producing the most biased estimates across most conditions due to exceptionally low recall rates. Its poor performance can be attributed to the mismatch between the algorithm’s mechanism and the latent structure of the data. While K-means is a partitioning method designed to identify distinct clusters, SOM focuses on topology preservation, attempting to map high-dimensional data onto a continuous grid. In this study, the difference between effortful and non-effortful (i.e., rapid guessing) behaviors represents a qualitative, discrete shift rather than a continuous transition. However, SOM likely blurred the separation between the two distinct classes during the topological mapping process, causing a large proportion of effortful respondents (particularly those with relatively high speed) to be misclassified into non-effortful nodes. Consequently, the severe loss of effective information (low recall) led to poor calibration accuracy. Given these findings, only K-means was retained for the main study.

This proposed method was compared with $l_{z}^{*}$ in terms of recall, precision, and *F*1. The recalls remained steady at over 96%. However, as indicated in the pre-study, K-means and SOM produced frustrating results (the recalls were < 50%), the high recall of the proposed method is primarily attributed to $l_{z}^{*}$. Turning to precision, the proposed method outperformed $l_{z}^{*}$ at the high-level severity. This is because K-means divides respondents based on their spatial locations. Non-effortful respondents respond to fewer items effortfully, a greater difference between them and effortful people. In this instance, it is easier to exclude non-effort respondents during the first identification.

In summary, the two-step method based on K-means is preferable when non-effort severity is high. This study confirms that K-means is indeed effective to some extent in identifying effortful respondents due to its unique advantage in precision. We suggest that researchers choose the two-step method in low-stakes assessments where people tend to respond to most items with little effort due to a lack of motivation. In this situation, the response patterns of non-effortful respondents are quite different from those of effortful respondents, resulting in a great distance between them. As a result, K-means can more easily and accurately distinguish the two types of respondents in low-stakes assessments than in high-stakes assessments, where individuals work hard to achieve high scores. It should be noted that the core idea of our approach can extend beyond any method with the assumption of known item parameters. In other words, it is promising to leverage K-means to improve other methods, such as the residual method (van der Linden & van Krimpen-Stoop, 2003) and forward search (Atkinson et al., 2004). The steps can be summarized as follows:

1. Use K-means to enact clustering (i.e., the first identification).
2. Estimate item parameters based on the effortful cluster.
3. Using the above estimates, compute the test statistics (with the assumption of known item parameters) for respondents.

This study is not free of limitations. First, we only focused on two common unsupervised learning algorithms to examine their identification patterns. And the two-step method based on K-means is not superior to $l_{z}^{*}$ when non-effort severity is low. Based on the crux of the improvement (i.e., accurate parameter estimation), future studies can combine more methods that require no strong assumptions with $l_{z}^{*}$. For example, latent class analysis and latent profile analysis can be helpful in the context of low-dimensional data (Y. Liu et al., 2020). Second, our findings are based on large sample sizes. Using the proposed method, the available sample size for item parameter estimation is roughly half of the total sample size. When the sample size is small or preserving statistical power is critical, it may be preferable to retain respondents with few non-effortful responses or remove responses rather than respondents. Therefore, it would be worthwhile to establish the optimal criteria for removing respondents and consider the removal of non-effortful responses in the first identification. Third, we only used response patterns and response times as the basis for identification. In the future, the first identification of the two-step method could be expanded to analyze accumulated log data, such as eye-tracking indicators (Man & Harring, 2022; Man et al., 2020). Since log data may provide extra information about response behaviors, and data mining techniques do well in multivariate analysis and do not require a detailed specification of variable relationships, adding such data could potentially improve the accuracy of the identification. Fourth, it is difficult to accurately measure each respondent’s severity in reality. Thus, researchers may struggle with determining whether the severity warrants adopting this method. To infer the severity, objective methods, such as inference based on the different shapes of response time distribution, should be developed. Furthermore, the threshold of the severity level still needs to be investigated through more thorough simulations. Fifth, the proposed method is essentially a noniterative procedure. While our results showed that this approach yielded satisfactory classification accuracy and parameter recovery. under most conditions, it is plausible that an iterative procedure (e.g., Patton et al., 2019) could further improve the estimation accuracy. Future research should systematically compare the proposed two-step method with such iterative approaches to investigate their respective applicability and computational efficiency. A limitation of the empirical example concerns the validation of the proposed method. Although this method yielded classifications that appeared more consistent with theoretical expectations than $l_{z}^{*}$, future research should utilize an experimental design or incorporate bogus items to conduct a rigorous validation, as responding processes vary across different non-effortful responding behaviors.

## Figures and Tables

**Table 1:** Simulation conditions.

Factor	Level	Setting
*I*	Low	500
	Medium	1000
	High	2000
*J*	Low	15
	Medium	30
	High	50
π	Low	20%
	High	40%
$\pi_{i}$	Low	$\pi_{i}\sim U\left(0,\;0.25\right)$
	High	$\pi_{i}\sim U\left(0.5,\;0.75\right)$
$d_{\mathrm{RT}}$	Low	$\log\left(t_{ij}\right)\sim N\left(-1,\;0.25\right)$ *
	High	$\log\left(t_{ij}\right)\sim N\left(-2,\;0.25\right)$ *

* $t_{ij}$ refers to the non-effortful response time.

**Table 2:** Parameter distributions.

Parameter	Distribution Setting	
$a_{j}$	*U* (1, 2.5)	
$b_{j}$	*N* (0, 1)	
$\alpha_{j}$	*U* (1.5, 2.5)	
$\beta_{j}$	*U* (−0.2, 0.2)	
$\xi_{i}={\left(\theta_{i},\tau_{i}\right)}^{\prime }$	High-speed	$N\;\left(\mu ,\;\Sigma \right)$, where $\mu ={\left(0,\;3.5\right)}^{\prime }$, $\Sigma =\left[\begin{array}{cc} 1 & 0.25\\ 0.25 & 0.1275 \end{array}\right]$
	Low-speed	$N\;\left(\mu ,\;\Sigma \right)$, where $\mu ={\left(0,\;-3.5\right)}^{\prime }$, $\Sigma =\left[\begin{array}{cc} 1 & 0.25\\ 0.25 & 0.1275 \end{array}\right]$

## Data Availability

The simulated data are available from the first author upon request. The empirical data used in this study is from: Patterns of Solution Behavior across Items in Low-Stakes Assessments, by Dena A. Pastor, Thai Q. Ong, and Scott N. Strickman, Educational Assessment, reprinted by permission of Informa UK Limited, trading Taylor & Francis Group, https://www.tandfonline.com.

## Note

1 The results of the number of effortful respondents identified by both methods are provided in our Supplementary Materials. The results of item parameter recovery based on the identified effortful groups are provided in our Supplementary Materials.

## Supplementary Materials

The following supporting information can be downloaded at: https://www.mdpi.com/article/10.3390/jintelligence14020030/s1, Table S1: *I* = 500: Item Parameter Recovery Results for Different Groups; Table S2: *I* = 1000: Item Parameter Recovery Results for Different Groups; Figure S1: *I* = 500, *J* = 15: The Number of Respondents Identified as Effortful Individuals by Two Methods; Figure S2: *I* = 500, *J* = 30: The Number of Respondents Identified as Effortful Individuals by Two Methods; Figure S3: *I* = 500, *J* = 50: The Number of Respondents Identified as Effortful Individuals by Two Methods; Figure S4: *I* = 1000, *J* = 15: The Number of Respondents Identified as Effortful Individuals by Two Methods; Figure S5: *I* = 1000, *J* = 30: The Number of Respondents Identified as Effortful Individuals by Two Methods; Figure S6: *I* = 1000, *J* = 50: The Number of Respondents Identified as Effortful Individuals by Two Methods; Figure S7: *I* = 2000, *J* = 15: The Number of Respondents Identified as Effortful Individuals by Two Methods; Figure S8: *I* = 2000, *J* = 30: The Number of Respondents Identified as Effortful Individuals by Two Methods; Figure S9: *I* = 2000, *J* = 50: The Number of Respondents Identified as Effortful Individuals by Two Methods; Figure S10: Absolute Bias of Discrimination and Difficulty Parameter Estimates Based on the Two-step Method and $l_{z}^{*}$; Figure S11: *MSE* of Discrimination and Difficulty Parameter Estimates Based on the Two-step Method and $l_{z}^{*}$.
