# Supplementary material for: A Two-Step Method Based on lz* for Identifying Effortful Respondents
Source: J Intell. 2026 Feb 13;14(2):30. doi: 10.3390/jintelligence14020030 (PMC12942148; doi:10.3390/jintelligence14020030)

Supplementary Material

Table S1. I = 500: Item Parameter Recovery Results for Different Groups.

Conditions				All the Respondents				The Effortful Group Identified by K-means				The Effortful Group Identified by SOM			
J	π	π_i	d_{RT}	|a|	|b|	a_MSE	b_MSE	|a|	|b|	a_MSE	b_MSE	|a|	|b|	a_MSE	b_MSE
15	20%	$U(0,0.25)$	$\log(t_{ij}) \sim N(-1,0.25)$	0.26	0.15	0.12	0.05	0.41	0.26	0.31	0.12	1.54	1.08	9.45	1.64
			$\log(t_{ij}) \sim N(-2,0.25)$	0.27	0.15	0.13	0.04	0.41	0.26	0.31	0.12	1.62	1.11	12.05	1.75
		$U(0.5,0.75)$	$\log(t_{ij}) \sim N(-1,0.25)$	0.56	0.42	0.67	0.31	0.57	0.32	0.67	0.17	1.77	0.93	17.08	1.26
			$\log(t_{ij}) \sim N(-2,0.25)$	0.59	0.43	0.75	0.32	0.59	0.31	0.71	0.17	2.03	0.95	21.38	1.28
	40%	$U(0,0.25)$	$\log(t_{ij}) \sim N(-1,0.25)$	0.31	0.17	0.15	0.05	0.45	0.30	0.34	0.16	1.76	1.12	14.48	1.90
			$\log(t_{ij}) \sim N(-2,0.25)$	0.31	0.18	0.15	0.06	0.44	0.30	0.35	0.16	1.83	1.12	17.07	1.71
		$U(0.5,0.75)$	$\log(t_{ij}) \sim N(-1,0.25)$	1.34	0.75	4.81	0.83	1.05	0.38	3.04	0.25	3.31	1.00	56.68	1.34
			$\log(t_{ij}) \sim N(-2,0.25)$	1.29	0.75	4.70	0.80	0.95	0.38	2.72	0.25	3.21	0.98	56.26	1.34
30	20%	$U(0,0.25)$	$\log(t_{ij}) \sim N(-1,0.25)$	0.25	0.14	0.11	0.04	0.37	0.26	0.26	0.13	0.88	0.90	2.92	1.14
			$\log(t_{ij}) \sim N(-2,0.25)$	0.26	0.15	0.12	0.04	0.38	0.26	0.27	0.13	0.87	0.91	2.94	1.13
		$U(0.5,0.75)$	$\log(t_{ij}) \sim N(-1,0.25)$	0.49	0.40	0.60	0.32	0.51	0.30	0.59	0.17	1.28	0.90	11.22	1.13
			$\log(t_{ij}) \sim N(-2,0.25)$	0.49	0.41	0.61	0.34	0.50	0.30	0.57	0.16	1.49	0.83	12.62	1.03
	40%	$U(0,0.25)$	$\log(t_{ij}) \sim N(-1,0.25)$	0.30	0.17	0.15	0.05	0.41	0.30	0.31	0.15	0.91	0.95	4.52	1.26
			$\log(t_{ij}) \sim N(-2,0.25)$	0.29	0.17	0.15	0.06	0.40	0.30	0.29	0.16	0.90	1.01	3.52	1.49
		$U(0.5,0.75)$	$\log(t_{ij}) \sim N(-1,0.25)$	1.32	0.75	6.83	0.85	0.85	0.41	2.52	0.27	1.98	0.93	29.25	1.33
			$\log(t_{ij}) \sim N(-2,0.25)$	1.08	0.70	4.44	0.80	0.81	0.40	1.87	0.27	1.96	0.91	31.42	1.25
50	20%	$U(0,0.25)$	$\log(t_{ij}) \sim N(-1,0.25)$	0.26	0.16	0.12	0.06	0.37	0.28	0.27	0.15	0.72	0.88	1.99	1.17
			$\log(t_{ij}) \sim N(-2,0.25)$	0.26	0.15	0.12	0.05	0.38	0.28	0.28	0.15	0.66	0.87	1.26	1.07
		$U(0.5,0.75)$	$\log(t_{ij}) \sim N(-1,0.25)$	0.55	0.40	1.02	0.38	0.51	0.29	0.64	0.16	1.04	0.81	7.30	1.02
			$\log(t_{ij}) \sim N(-2,0.25)$	0.55	0.39	0.97	0.34	0.52	0.29	0.67	0.16	1.28	0.81	13.06	1.03

Conditions				All the Respondents				The Effortful Group Identified by K-means				The Effortful Group Identified by SOM			
J	π	π_i	d_{RT}	|a|	|b|	a_*MSE*	b_*MSE*	|a|	|b|	a_*MSE*	b_*MSE*	|a|	|b|	a_*MSE*	b_*MSE*
50	40%	$U\,(0,0.25)$	$\log(t_{ij}) \sim N(-1,0.25)$	0.30	0.18	0.16	0.07	0.40	0.30	0.30	0.16	0.86	0.88	3.90	1.18
			$\log(t_{ij}) \sim N(-2,0.25)$	0.30	0.19	0.16	0.07	0.42	0.31	0.32	0.19	0.81	0.90	2.84	1.40
		$U\,(0.5,0.75)$	$\log(t_{ij}) \sim N(-1,0.25)$	1.61	0.68	15.07	0.78	0.76	0.39	1.47	0.25	1.46	0.82	11.57	0.99
			$\log(t_{ij}) \sim N(-2,0.25)$	1.32	0.68	8.59	0.77	0.84	0.39	3.16	0.27	1.54	0.86	21.47	1.21

Note. |a|: the absolute bias of the discrimination parameter; |b|: the absolute bias of the difficulty parameter; a_*MSE*: the mean squared error of the discrimination parameter; b_*MSE*: the mean squared error of the difficulty parameter.

Table S2. I = 1000: Item Parameter Recovery Results for Different Groups.

Conditions				All the Respondents				The Effortful Group Identified by K-means				The Effortful Group Identified by SOM			
J	π	π_i	d_{RT}	|a|	|b|	a_*MSE*	b_*MSE*	|a|	|b|	a_*MSE*	b_*MSE*	|a|	|b|	a_*MSE*	b_*MSE*
15	20%	$U(0,0.25)$	$\log(t_{ij}) \sim N(-1,0.25)$	0.20	0.10	0.07	0.02	0.29	0.21	0.13	0.08	1.03	1.19	3.70	1.78
			$\log(t_{ij}) \sim N(-2,0.25)$	0.20	0.10	0.07	0.02	0.28	0.21	0.13	0.07	0.88	1.14	1.72	1.60
		$U(0.5,0.75)$	$\log(t_{ij}) \sim N(-1,0.25)$	0.42	0.40	0.36	0.27	0.34	0.25	0.22	0.10	0.85	1.10	2.40	1.52
			$\log(t_{ij}) \sim N(-2,0.25)$	0.41	0.40	0.33	0.27	0.34	0.25	0.22	0.09	0.90	1.13	2.40	1.55
	40%	$U(0,0.25)$	$\log(t_{ij}) \sim N(-1,0.25)$	0.26	0.13	0.12	0.03	0.33	0.24	0.17	0.09	0.91	1.19	2.05	1.76
			$\log(t_{ij}) \sim N(-2,0.25)$	0.26	0.12	0.11	0.03	0.32	0.23	0.17	0.09	1.02	1.18	4.12	1.85
		$U(0.5,0.75)$	$\log(t_{ij}) \sim N(-1,0.25)$	0.96	0.72	2.03	0.73	0.47	0.34	0.46	0.18	1.49	1.10	9.49	1.48
			$\log(t_{ij}) \sim N(-2,0.25)$	0.94	0.72	2.02	0.73	0.48	0.33	0.46	0.17	1.29	1.08	7.05	1.41
30	20%	$U(0,0.25)$	$\log(t_{ij}) \sim N(-1,0.25)$	0.19	0.11	0.07	0.02	0.26	0.21	0.12	0.08	0.61	1.02	0.98	1.38
			$\log(t_{ij}) \sim N(-2,0.25)$	0.19	0.11	0.06	0.02	0.26	0.21	0.12	0.08	0.59	1.12	0.76	1.73
		$U(0.5,0.75)$	$\log(t_{ij}) \sim N(-1,0.25)$	0.38	0.39	0.34	0.28	0.29	0.25	0.16	0.10	0.62	1.04	0.84	1.42
			$\log(t_{ij}) \sim N(-2,0.25)$	0.38	0.39	0.34	0.30	0.29	0.25	0.16	0.10	0.58	1.00	0.85	1.22
	40%	$U(0,0.25)$	$\log(t_{ij}) \sim N(-1,0.25)$	0.25	0.14	0.12	0.04	0.30	0.24	0.16	0.10	0.60	1.05	0.73	1.64
			$\log(t_{ij}) \sim N(-2,0.25)$	0.25	0.14	0.12	0.04	0.30	0.24	0.16	0.10	0.65	1.07	1.19	1.48
		$U(0.5,0.75)$	$\log(t_{ij}) \sim N(-1,0.25)$	0.85	0.69	2.13	0.72	0.43	0.34	0.36	0.18	0.91	0.98	2.62	1.30
			$\log(t_{ij}) \sim N(-2,0.25)$	0.90	0.70	2.20	0.75	0.43	0.34	0.36	0.18	0.97	1.02	5.19	1.36
50	20%	$U(0,0.25)$	$\log(t_{ij}) \sim N(-1,0.25)$	0.19	0.12	0.07	0.03	0.25	0.21	0.11	0.08	0.49	0.94	0.60	1.21
			$\log(t_{ij}) \sim N(-2,0.25)$	0.19	0.11	0.07	0.03	0.25	0.22	0.11	0.08	0.52	0.90	0.60	1.10
		$U(0.5,0.75)$	$\log(t_{ij}) \sim N(-1,0.25)$	0.42	0.38	0.46	0.31	0.28	0.26	0.16	0.11	0.54	0.97	0.70	1.18
			$\log(t_{ij}) \sim N(-2,0.25)$	0.40	0.36	0.44	0.30	0.29	0.25	0.16	0.11	0.56	0.98	0.67	1.24

Conditions				All the Respondents				The Effortful Group Identified by K-means				The Effortful Group Identified by SOM			
J	π	π_i	d_{RT}	|a|	|b|	a_*MSE*	b_*MSE*	|a|	|b|	a_*MSE*	b_*MSE*	|a|	|b|	a_*MSE*	b_*MSE*
50	40%	$U(0,0.25)$	$\log(t_{ij}) \sim N(-1,0.25)$	0.25	0.15	0.12	0.04	0.30	0.25	0.16	0.11	0.54	1.00	0.68	1.49
			$\log(t_{ij}) \sim N(-2,0.25)$	0.25	0.14	0.12	0.04	0.30	0.25	0.16	0.11	0.59	1.06	0.94	1.48
		$U(0.5,0.75)$	$\log(t_{ij}) \sim N(-1,0.25)$	0.78	0.64	2.03	0.66	0.42	0.33	0.38	0.18	0.82	0.99	3.14	1.37
			$\log(t_{ij}) \sim N(-2,0.25)$	0.83	0.66	2.21	0.70	0.43	0.34	0.44	0.19	0.81	0.92	2.70	1.06

Note. |a|: the absolute bias of the discrimination parameter; |b|: the absolute bias of the difficulty parameter; a_*MSE*: the mean squared error of the discrimination parameter; b_*MSE*: the mean squared error of the difficulty parameter.

Figure S1. $I = 500$, $J = 15$: The Number of Respondents Identified as Effortful Individuals by Two Methods.

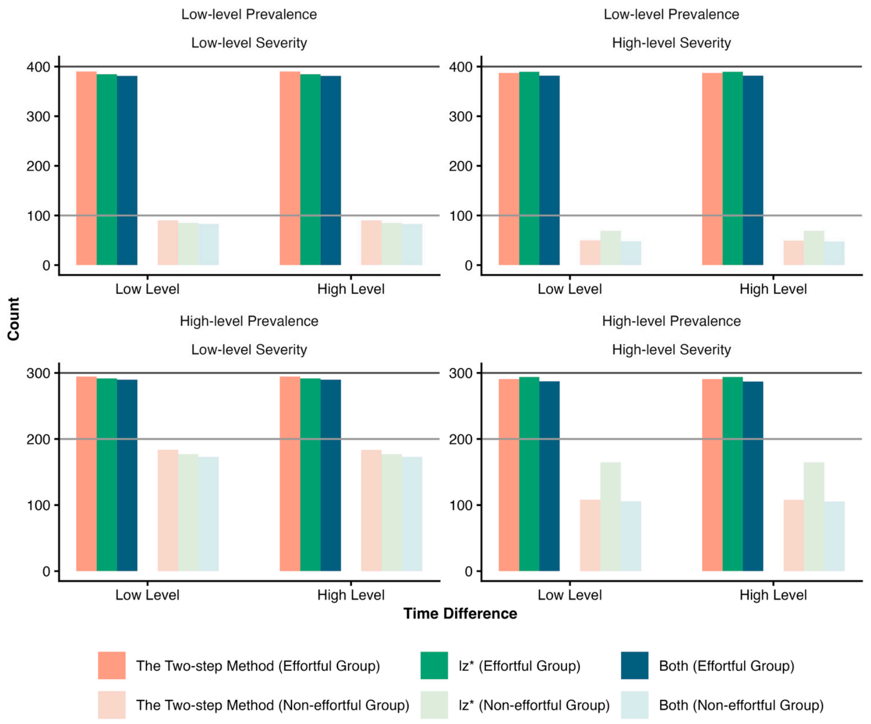

Figure S2. $I = 500$, $J = 30$: The Number of Respondents Identified as Effortful Individuals by Two Methods.

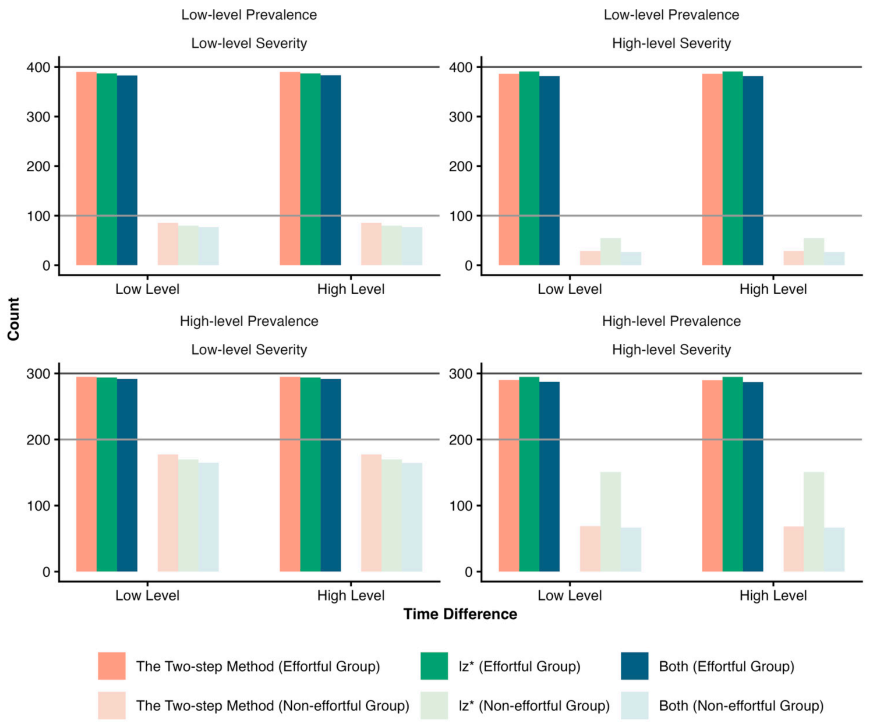

Figure S3. $I = 500$, $J = 50$: The Number of Respondents Identified as Effortful Individuals by Two Methods.

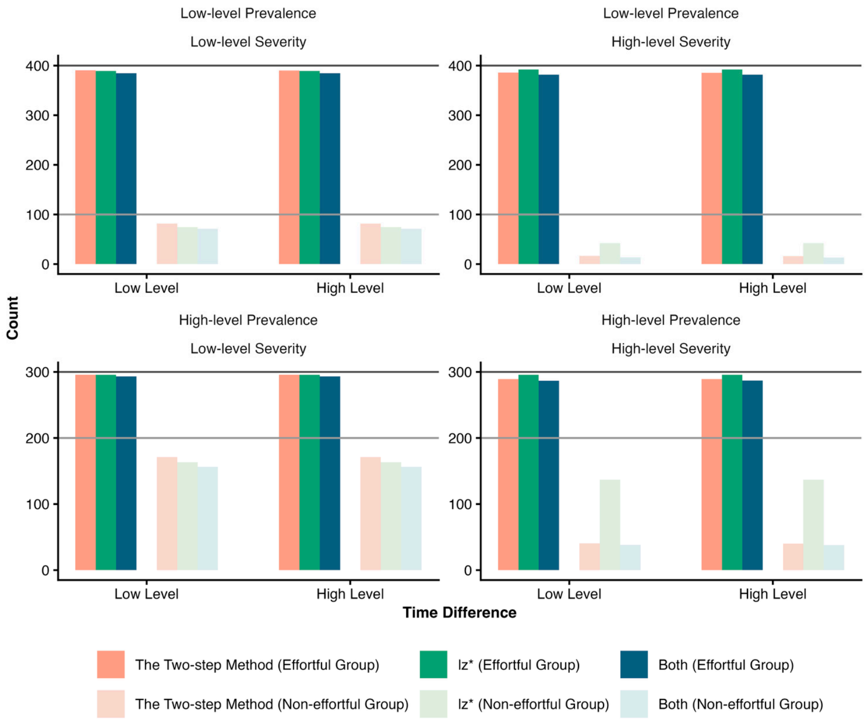

Figure S4. $I = 1000$, $J = 15$: The Number of Respondents Identified as Effortful Individuals by Two Methods.

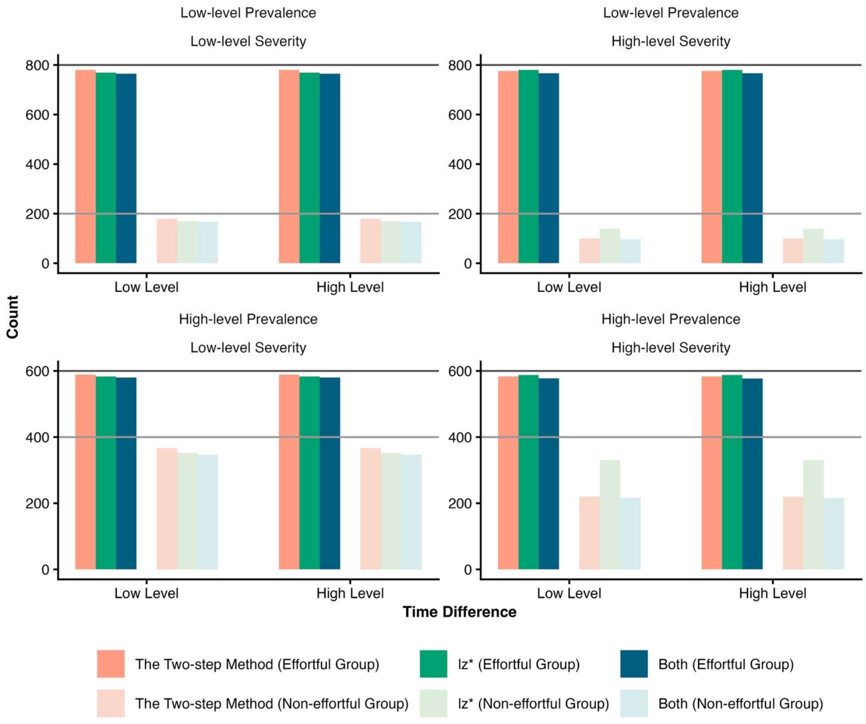

Figure S5. $I = 1000$, $J = 30$: The Number of Respondents Identified as Effortful Individuals by Two Methods.

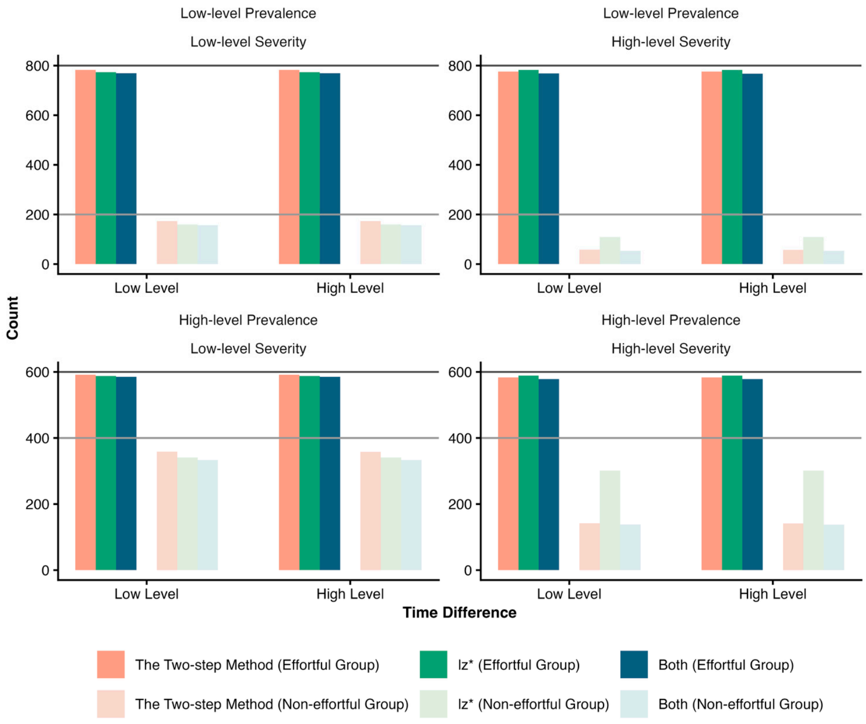

Figure S6. $I = 1000$, $J = 50$: The Number of Respondents Identified as Effortful Individuals by Two Methods.

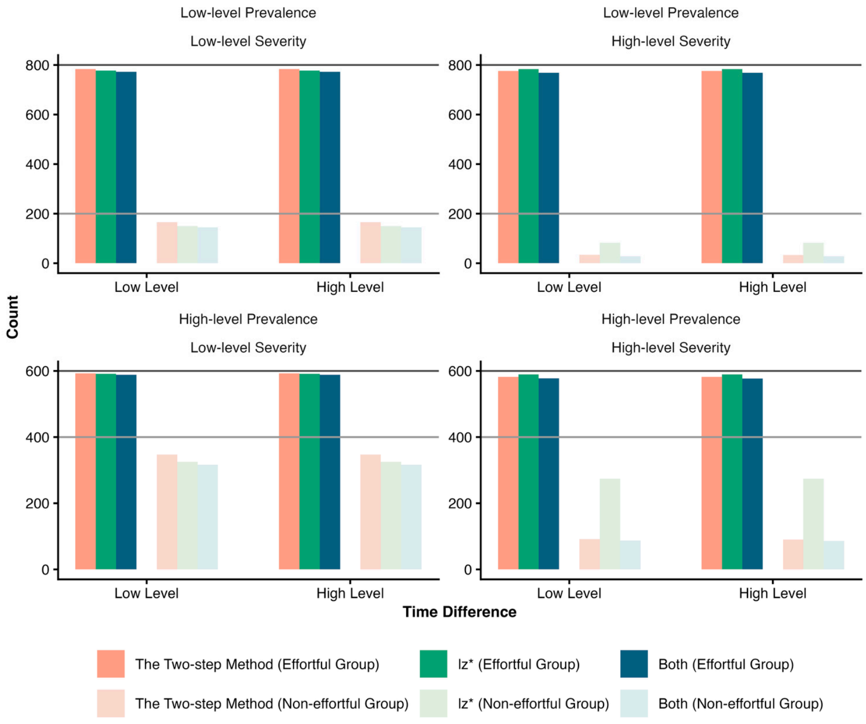

Figure S7. $I = 2000$, $J = 15$: The Number of Respondents Identified as Effortful Individuals by Two Methods.

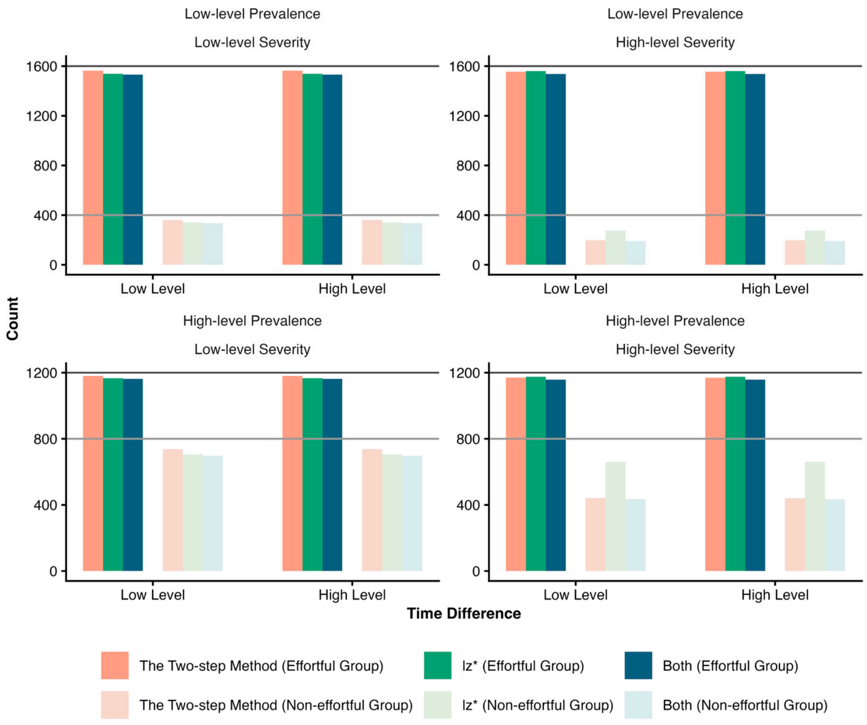

Figure S8. $I = 2000$, $J = 30$: The Number of Respondents Identified as Effortful Individuals by Two Methods.

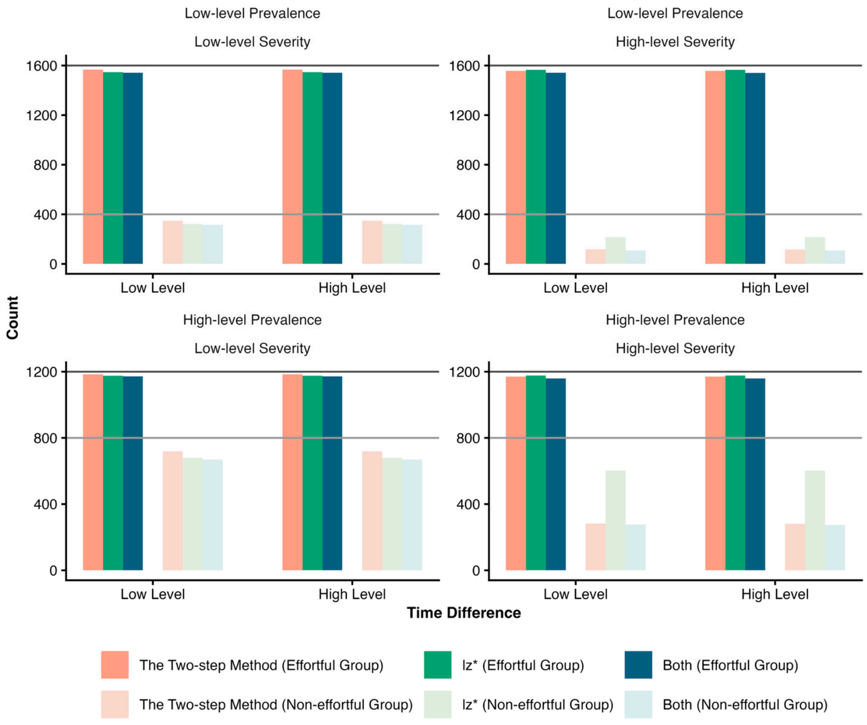

Figure S9. $I = 2000$, $J = 50$: The Number of Respondents Identified as Effortful Individuals by Two Methods.

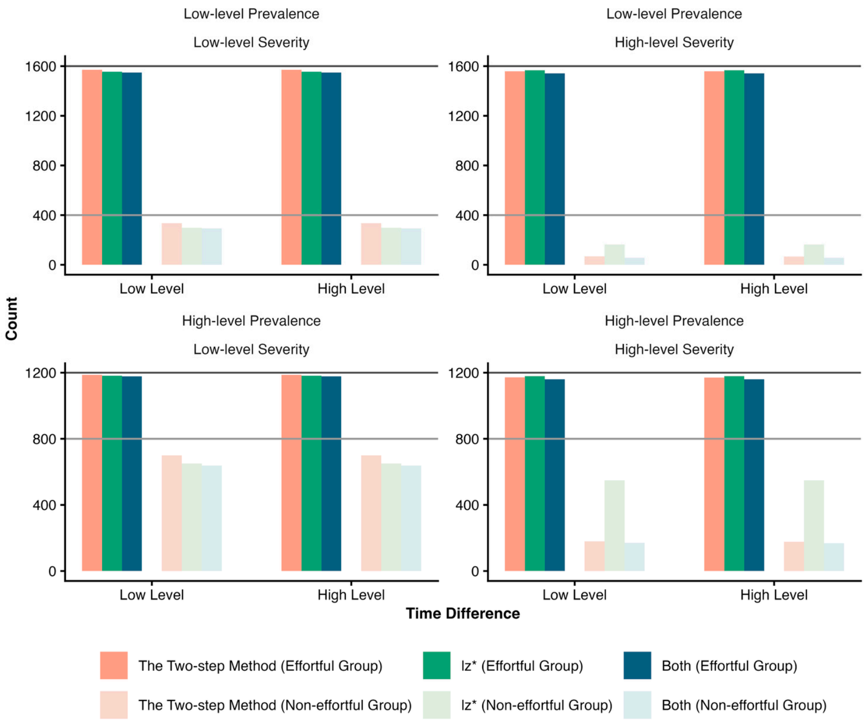

Figure S10. Absolute Bias of Discrimination and Difficulty Parameter Estimates Based on the Two-step Method and l_z^*.

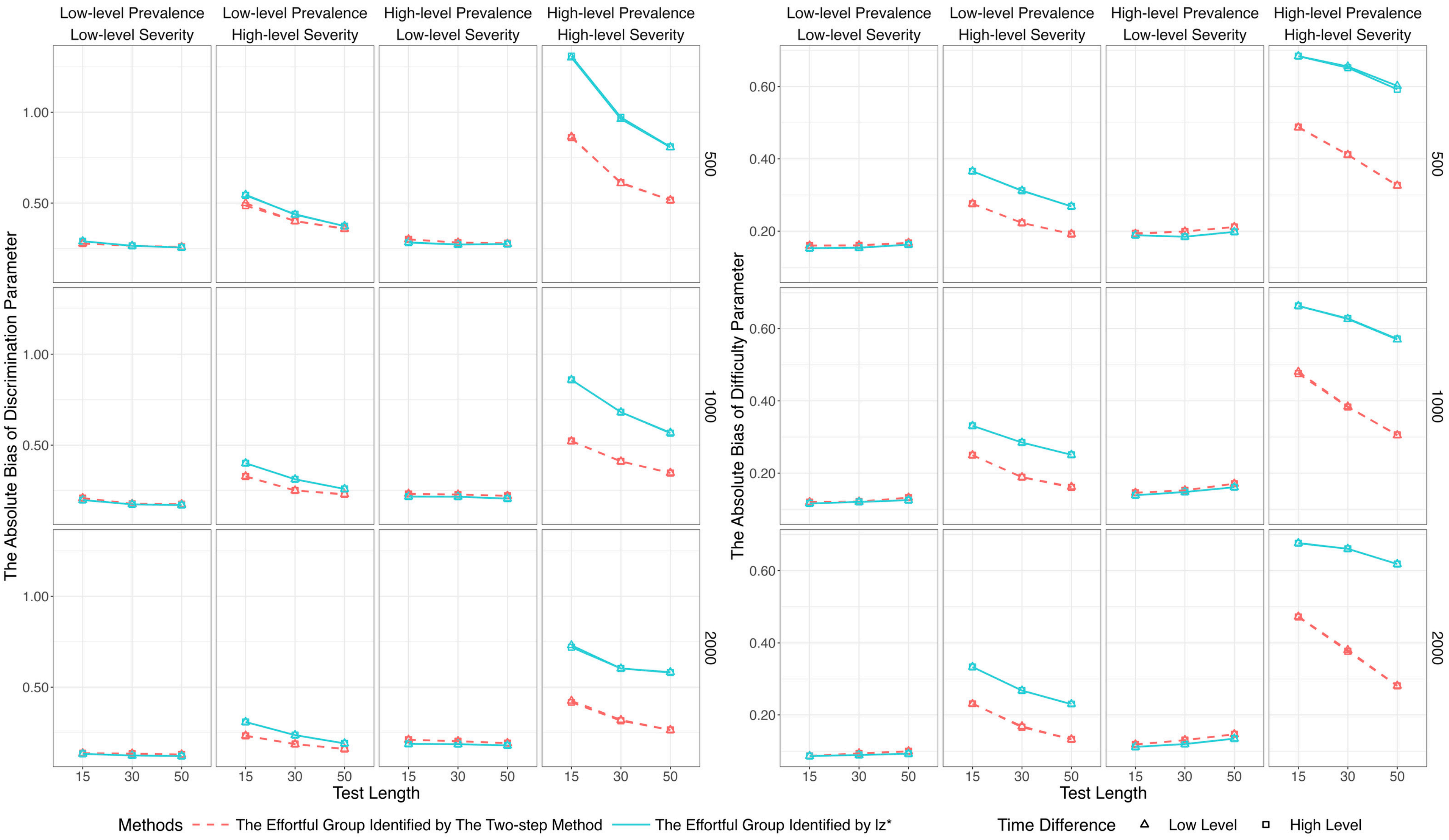

Figure S11. *MSE* of Discrimination and Difficulty Parameter Estimates Based on the Two-step Method and l_z^*.

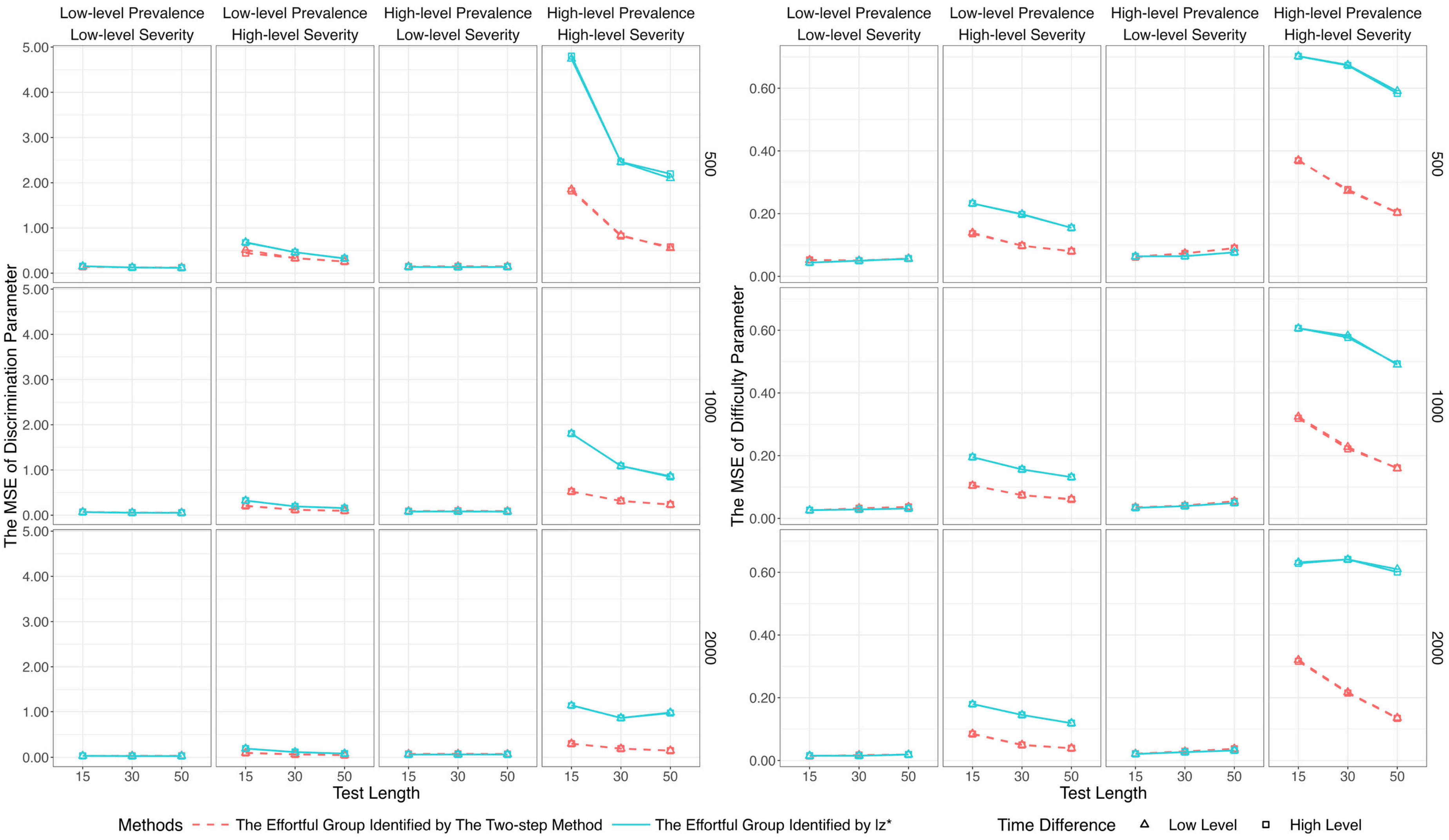

Supplement: Supplementary file 1 [file jintelligence-14-00030-s001.zip › jintelligence-3932246-supplementary.pdf]
